# Patient-centered Pharmacist Care in the Hemodialysis Unit: a quasi-experimental interrupted time series study

**DOI:** 10.1186/s12882-019-1577-6

**Published:** 2019-11-13

**Authors:** Sherine Ismail, Abrar Al-Subhi, Eman Youssif, Medhat Ahmed, Abdullah Almalki, Diane L. Seger, Andrew C. Seger, Earl Cook

**Affiliations:** 1 King Abdullah International Medical Research Center, King Saud bin Abdulaziz University for Health Sciences, Pharmaceutical Care Department, King Khalid Hospital, Ministry of National Guard Health Affairs, Jeddah, Saudi Arabia; 2 Harvard T. H. Chan School of Public Health, Boston, MA USA; 3 King Abdullah International Medical Research Center, King Saud bin Abdulaziz University for Health Sciences, Medicine Department, Nephrology Section, King Khalid Hospital, Ministry of National Guard Health Affairs, Jeddah, Saudi Arabia; 4 Partners Healthcare System, Somerville, MA USA; 5 Brigham and Women’s Hospital, Boston, MA USA; 6 Harvard T. H. Chan School of Public Health, Boston, MA, United States, Brigham and Women’s Hospital, Boston, MA USA

**Keywords:** Patient-centered pharmacist care, Adherence, Pharmacoadherence, Hemodialysis, Medication-related problems, Medication therapy management, Motivational interview

## Abstract

**Background:**

Nonadherence to medications by patients requiring hemodialysis (HD) leads to unfavorable clinical outcomes. Limited data exist to demonstrate the effect of incorporating patient-centered interventions using concepts of medication therapy management and motivational interview by pharmacists on pharmacoadherence in patients requiring HD. Therefore, we assessed the impact of patient-centered pharmacist care on pharmacoadherence and its outcomes in patients requiring HD.

**Methods:**

Adult patients who had received outpatient HD for at least 3 months were enrolled. The study was conducted from October 2016 to April 2017. Pharmacists interviewed the patients at month 1, 2, 4 and 6, and the intervention (comprehensive review) occurred at months 3 and 5. The primary outcome was the change in pharmacoadherence as assessed by pre-HD serum phosphate levels and the differences in the number of medications between patient’ self-report and medications records at the electronic healthcare records (EHRs). The secondary outcomes included changes in systolic blood pressure (SBP), glycosylated hemoglobin levels, serum low-density lipoprotein (LDL) levels, and the prevalence and types of medication-related problems (MRPs).

**Results:**

Seventy-two patients were enrolled. Their median age was 59 (interquartile range: 47–67.5) years, and 53% were men. Pre- and post-intervention pharmacoadherence, as indicated by serum phosphate levels and the differences in the number of medications between patient’ self-report and the medication records at the EHRs, did not significantly differ (*p* = 0.682 and 0.348, respectively). Mean SBP and mean LDL did not significantly change post-intervention. The median number of MRPs declined between Months 3 and 5 (*p* = 0.002): the prevalence of MRPs at Month 3 was 44.9% (95 confidence interval [CI]: 40.4–49.3) and decreased to 29.8% (95 CI: 25.6–34.3) at Month 5. Drug use without indication was the most frequent MRP (23.9%).

**Conclusions:**

Patient-centered pharmacist care did not result in significant changes in pharmacoadherence. However, its clinical utility as a tool to identify and mitigate MRPs in patients requiring HD is indisputable.

**Trial Registration:**

ClinicalTrials.gov identifier: NCT03576404 (retrospectively registered on July 3rd, 2018).

## Background

In 2003, the World Health Organization (WHO) announced that nonadherence to medications is a public health problem among patients with chronic illnesses that leads to unfavorable clinical outcomes and confers a significant financial burden on medical institutions [1]. Furthermore, the WHO proposed that the optimization of pharmacoadherence [2] should serve as a modifier of the effectiveness of the health-care system, improving patient safety and health.

Considerable variation in the rate of nonadherence to medications, which ranges from 12.5–98.6%, has been reported in patients requiring hemodialysis (HD) [3]. This variation has been attributed to the different measurement tools used, which include:

(i) serum drug concentrations or biologic tracers (direct);
(ii) electronic prescribing, refill records, and pill counts, or surrogate clinical outcomes such as pre-HD phosphate levels (indirect);
(iii) self-reported patient questionnaires or diaries or assessment by clinicians (subjective); and
(iv) combined strategies [2, 3].

Although hyperphosphatemia is a common medical problem in patients requiring HD, low and high serum phosphate levels are significantly associated with increased all-cause mortality and cardiovascular mortality in patients requiring HD [4]. The mean rate of adherence to phosphate binders by patients requiring HD is reportedly 51% (range: 22–74%) [5, 6]. Therefore, several studies included in a systematic review by Karamanidou et al. have utilized adherence to phosphate binders and serum phosphate levels as surrogate markers of adherence in patients requiring HD [5].

Likewise, the administration of antihypertensive agents compared to the control arms (which included placebo or conventional treatment) in patients requiring HD in a meta-analysis of eight RCTs was associated with improved outcomes such as reduced risks of cardiovascular events, all cause mortality and cardiovascular mortality [7]. Furthermore, there is an inverse association between the number of comorbidities and adherence to antihypertensive agents [8]. Patients requiring HD are prone to several chronic comorbidities, which necessitate the use of several medications and hence are at high risk of nonadherence. Subsequently, the prevalence of polypharmacy among patients requiring HD is high, which leads to medication-related problems (MRPs) [9, 10].

Several studies have identified factors that influence pharmacoadherence in patients requiring HD, such as demographic, clinical, and psychosocial factors [11, 12]. As a result of the multidimensional nature of pharmacoadherence in patients requiring HD, many interventions have been proposed to improve adherence to medications in this population. One such intervention focuses on the beliefs of patients regarding their disease and medications, improving health literacy and emphasizes the role of the renal pharmacist as a core member of the multidisciplinary team that cares for patients requiring HD [13–17]. Similarly, medication therapy management (MTM) and motivational interviewing (MI) are patient-centered approaches that provide unique pharmacy practice models to improve pharmacoadherence and optimize therapeutic outcomes in patients with several chronic illnesses [18–21].

Medication management has been identified as one of the surrogate markers of higher complex programs located in the middle of the patient-centered quality hierarchy (quality pyramid) for the care for patients requiring HD [22]. The fundamental care for patients requiring HD such as achieving target indicators for anemia management and adequacy of dialysis recognized as the base of the pyramid whereas health-related quality of life was the ultimate goal at the top of the pyramid [22]. Several reports pointed to the successful role of pharmacists as a member of multidisciplinary team to achieve the fundamental care for patients requiring HD [23–25].

To date, limited data exist on successful patient-centered interventions conducted by pharmacists using concepts of MTM and MI to improve pharmacoadherence in patients requiring HD. Therefore, we assessed the impact of patient-centered pharmacist care by performing a comprehensive medication review using concepts of MTM and MI to improve pharmacoadherence in patients requiring HD.

## Methods

### Study design and setting

We conducted a prospective study with a quasi-experimental, interrupted time series design [26]. The study took place between October 2016 and April 2017 at the outpatient HD unit of the King Abdulaziz Medical City, Jeddah, Saudi Arabia.

### Eligibility criteria

We included adult patients (≥18 years of age) who had received outpatient HD for at least 3 months before the study period. Patients who refused to participate in the study and those without the capacity to understand or take responsibility for their medications were excluded. We randomly assigned 72 unique computer-generated table numbers for the eligible participants [27].

### Baseline characteristics

Baseline demographics, educational level and medication-related factors such as pill burden, total number of tablets per day were identified through patient’s interview and using the electronic healthcare records (EHRs). In addition, clinical factors, comorbidities and dialysis-related factors were recorded as documented in the EHRs by various healthcare providers.

### Outcomes

The primary outcome of the study was to assess the impact of a patient-centered pharmacist care on the pharmacoadherence of patients requiring HD using the changes in serum phosphate levels and differences in number of medications between patient’ self-report and medication records at the EHRs.

Secondary outcomes included intermediate outcomes such as systolic blood pressure (SBP) control, serum low-density lipoprotein (LDL) levels among patients receiving lipid-lowering agents, and glycosylated hemoglobin (HbA1c) levels for patients with diabetes. We also determined the prevalence and types of MRPs in our cohort. Other secondary outcomes included the therapeutic interventions suggested by pharmacists, the proportions of which were accepted or rejected by physicians, and the reasons for rejection if rejected.

### Intervention and follow-up assessment

**Fig. 1:**
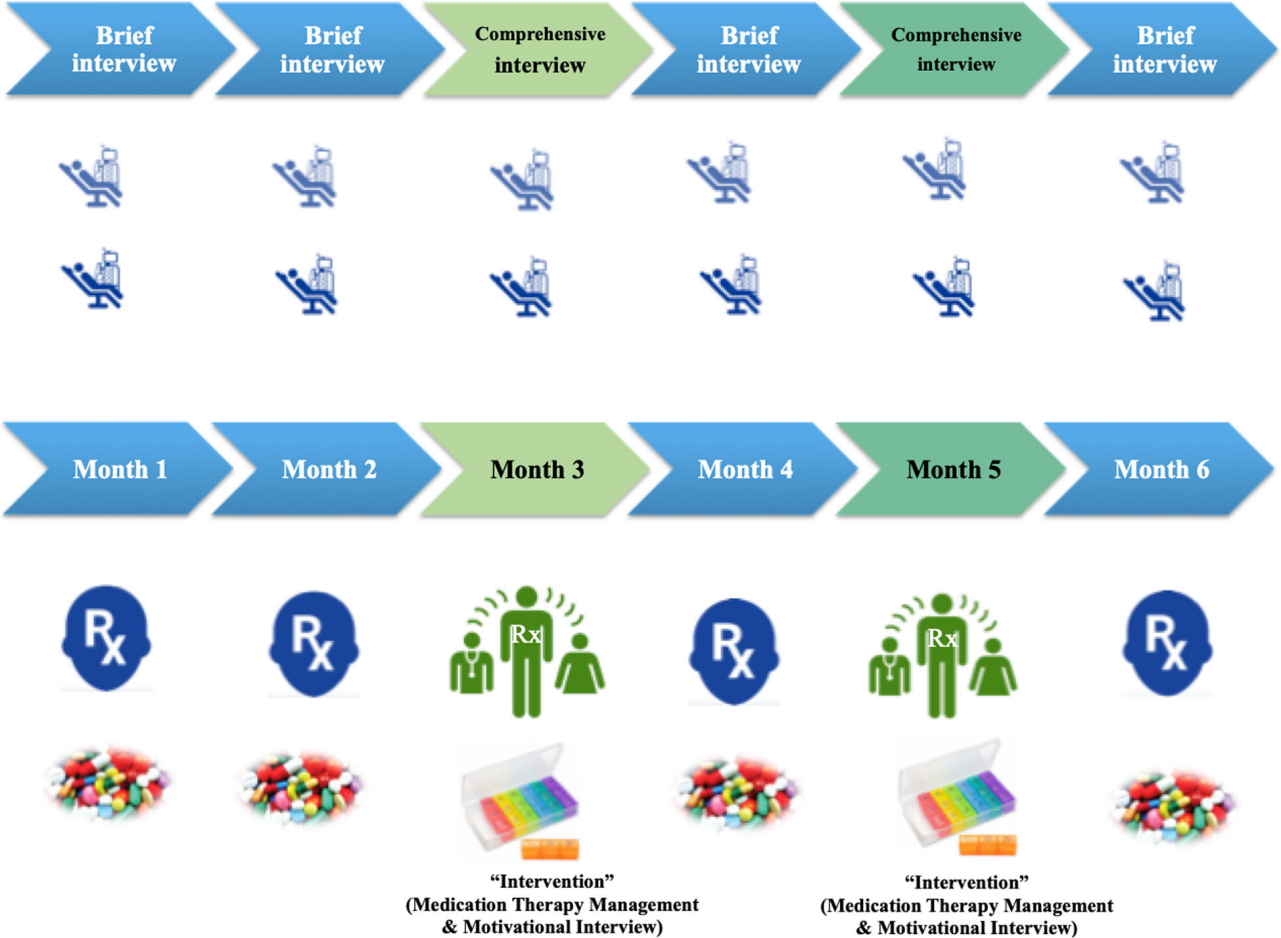
Study layout. The blood pressure and laboratory parameters are recorded on monthly basis during those 6 months and one additional month afterwards

The intervention was the patient-centered pharmacist care, which was defined as the comprehensive medication review using concepts of MTM and MI. A team of four pharmacists interviewed patients or their caregivers who participated in the administration of medications on month 1, 2, 4 and 6 to assess the self-reporting of medications and compare it with electronic prescribing from October 2016–March 2017. One clinical pharmacist of the four pharmacists conducted a comprehensive, structured review of the medications of each participant at Months 3 and 5 through a face-to-face interview using MTM and MI (Fig. 1). Each patient at Months 1, 2, and 3 (pre-intervention) served as his/her own control for observations at Months 4, 5, and 6 (post-intervention).

MTM included a review of each participant’s medications and personal medication record to identify the use of any over-the-counter medications, design of their medication-related action plan, formulation of specific therapeutic interventions, and referrals for discussion with their physician and subsequent documentation and follow-up. The therapeutic interventions accepted by the physician which warrant changes in therapeutic regimens were further conveyed to each patient/caregiver using the MI technique to encourage adherence and documented in each patient’s medication record. MI techniques used in our study incorporated the main components reported in the literature: rolling with resistance; expressing empathy; avoiding argumentative behavior; highlighting the discrepancy between patients’ current attitudes compared with the desired therapeutic goals; and empowering patients’ self-efficacy [20, 28, 29]. MI started at Month 3 during the comprehensive interview and continued until the end of the study, whenever a new medication was prescribed or as deemed clinically necessary, during the follow-up period. The aim of MI was to empower patients with necessary information about their diseases, address the beliefs about their medications and barriers for non-adherence, and provide specific instructions to optimize the use and administration of each medication on individual basis. The study participants and /or care givers also received pillboxes to assist with their pharmacoadherence at Month 3 and an Arabic written medication list based on the action plan developed in partnership with their health-care providers by Month 4. Participants whose therapeutic regimens changed received an updated list of medications by Months 5–6.

### The standard of care

The standard of care in the unit was the presence of the same clinical pharmacist participating in multidisciplinary monthly rounds, making therapeutic recommendations to the team and counseling patients regarding changes in their medications and as deemed necessary by referrals. It was continuous throughout the study period.

### Outcome assessment

To assess the primary outcome of pharmacoadherence, we compared patients’ self-reporting of medications with electronic prescribing during each of the six interviews and another indirect measure of pharmacoadherence, surrogate laboratory pre-HD serum phosphate level, on a monthly basis. Secondary outcomes, such as surrogate HbA1c levels among patients with diabetes, SBP, and LDL levels among patients receiving lipid-lowering agents, were recorded before and after the intervention. SBP readings for every month of the study period were obtained as an average of three consecutive pre-HD SBP readings. All surrogates laboratory parameters and SBP were recorded monthly for seven-time points; from October 2016 to December 2016 to present the pre-phase (three-time points) and from January to April 2017 to reflect the post-phase (four-time points). The MRPs included in the study as reported in the literature were: improper drug dosing or selection; initiation of medication without indication; adverse drug events; failure to receive drugs; indication without treatment; suggested alternative therapeutic options; and inappropriate monitoring or laboratory tests required [10].

### Sample size

In the absence of information on the variability of our primary outcomes, sample size estimation was calculated based on a binary measure for non-adherence rate to medications of 72% in hemodialysis patients who were dialyzing in a similar setting to our center [30].

We estimated that a sample of at least 60 patients would be sufficient to detect a 25% improvement in adherence due to our intervention based on clinical judgment, with a power of 80% and an α value of 0.05 (StataCorp LP, College Station, TX, USA).

### Statistical analysis

We used descriptive statistics for baseline characteristics, comorbidities, MRPs and their types, and suggested interventions. Linear mixed random segmented regression analysis was used to analyze the mean changes in surrogate end points (phosphate levels, SBP, HbA1c levels in patients with diabetes, and LDL levels among patients receiving lipid-lowering agents) and differences between self-reported medication use and electronic prescribing [31, 32]. The model assessed the changes in the surrogate endpoints by measuring the differences in the slope of the fitted line presenting the months before the intervention and slope of the fitted line presenting the months after the intervention and subsequently we assessed if these changes were significant or not [28]. The prevalence of MRPs was calculated by dividing the total number of MRPs by the total number of prescribed medications; 95% confidence intervals (CIs) were generated using the binomial exact test. We used the Wilcoxon signed-rank test to compare the median difference in the number of MRPs per prescribed medication at Months 3 (baseline) and 5 (after intervention). Two-sided tests were used for all analyses, and a *p* value < 0.05 was considered to indicate statistical significance. We conducted all analyses using STATA 2014 (StataCorp LP, College Station, TX, USA).

## Results

**Fig. 2:**
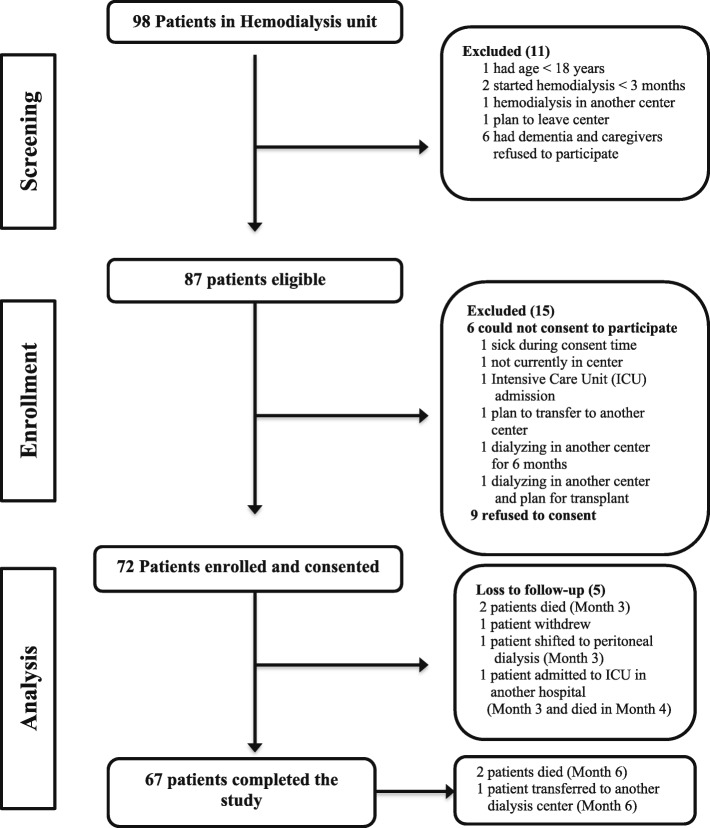
Trial profile: flow of participants through the trial

We screened 98 patients requiring HD, of whom 87 were eligible and 72 were successfully enrolled into the study. Figure 2 presents the trial profile and flow of study participants. The median age of the participants was 59 (interquartile range [IQR]: 47–67.5) years, and 53% were men. One-third of the patients had caregivers, of whom 50% were their daughters, 58% provided care most of the time and 83% of the caregivers had level of education of elementary or above. The mean pill burden identified from patients’ electronic records was 11.3 (standard deviation: 5.5), of which patients’ self-reported a median daily tablet count of seven (IQR: 5–11) in the first month of the study. Other baseline demographic characteristics of the patients that may influence pharmacoadherence, such as clinical, HD-related, and medication-related factors, are illustrated in Table 1.

**Table 1:** Baseline characteristics

Baseline characteristics ^a^	Patients (*n* = 72)
**Patient factors**	
Age (years)	59 (47–67.5)
Sex (male)	38 (52.8)
Body mass index (kg/m^2^)	24.8 (21.7–29.8)
Educational level	
Illiterate	30 (41.7)
Preparatory school and below	25 (34.7)
High school and above	17 (23.6)
Marital status (married)	55 (76.4)
Employed	12 (16.7)
Smoking^b^	7 (9.7)
Caregiver	24 (33.3)
**Clinical factors**	
Longevity on dialysis (> 5 years)	31 (43)
Recent hospitalization	8 (11.1)
Diabetes	38 (52.8)
Hypertension	65 (90.3)
Ischemic heart disease	16 (22.2)
Heart failure^c^	4 (5.6)
Cerebrovascular infarction	10 (13.9)
Peripheral vascular disease^c^	9 (12.5)
Atrial fibrillation	5 (6.9)
Dementia	2 (2.8)
Previous history of kidney transplant	2 (2.8)
Hepatitis B	4 (5.6)
Hepatitis C	8 (11.1)
Parathyroid adenoma	7 (10%)
**Dialysis-related factors**	
Access of dialysis: arteriovenous fistula	33 (45.8)
Adequacy of dialysis: (Kt/V) ^c^	1.58 ± 0.33
Intra-dialytic weight gain to dry body weight percentage	3.4 (2.5–5)
Pre-dialysis phosphate (mmol/L)	1.45 (1.5–1.8)
Intact parathyroid hormone (pg/mL)	545 (361–1236)
**Medication-related factors**	
Total pill burden (electronic records)	11.3 ± 5.5
Daily tablet count (self-reported)	7 (5–11)
Number of medications per patient (electronic records)	7 (5–8)

Values are given as *n* (%), mean ± standard deviation, or median (interquartile range). ^a^ All baseline characteristics were measured in the first month of the study except for the adequacy of dialysis, which is the mean of the first three months. All baseline characteristics were based on the documentation in EHRs and patients’ self-reports. ^b^ Smoking: the data for one patient was missing.^c^ As reported in the electronic healthcare records. Kt/V: a measure for assessing the adequacy of dialysis based on the clearance of urea during dialysis

**Fig. 3:**
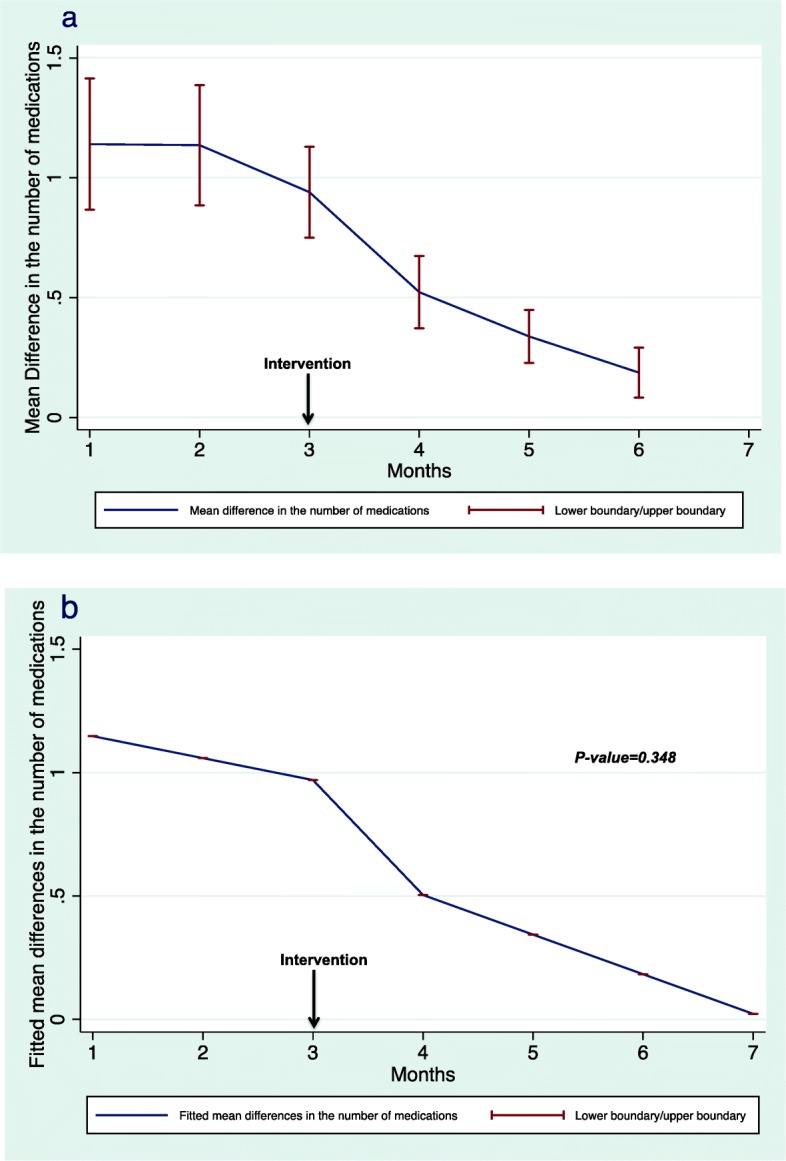
Mean (**a**) and fitted mean (**b**) differences in the number of medications . 3a. The mean difference between self-reported medication use and medications records at the electronic healthcare records identified during the six interviews. 3b.The fitted mean resulting from linear mixed regression analysis demonstrating the fitted mean before and after the intervention with a model prediction for the seventh month

**Fig. 4:**
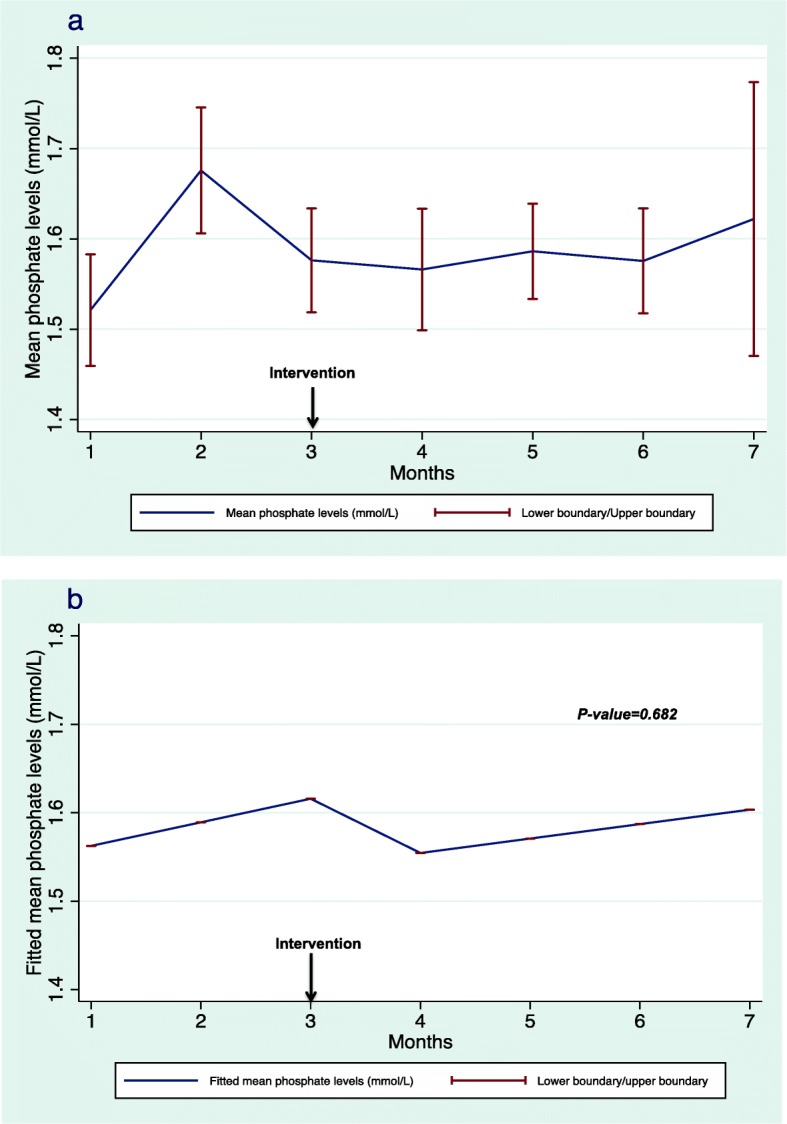
Mean (**a**) and fitted mean (**b**) phosphate levels. The fitted mean resulting from linear mixed regression analysis demonstrating the fitted mean before and after the intervention

The mean difference between self-reported medication use and electronic prescribing was reduced after the intervention. However, this change in pharmacoadherence was not statistically significant (*p* = 0.348). Figure 3a illustrates the change in the mean difference between self-reported medication use and electronic records identified during the six interviews, whereas Fig. 3b demonstrates the fitted mean differences before and after the intervention with a prediction for the seventh month by the model. Mean pre-HD phosphate levels decreased after the intervention, but without statistical significance (*p* = 0.682). Figure 4a illustrates the changes in mean pre-HD phosphate levels, and Fig. 4b demonstrates the fitted mean differences before and after the intervention. The results of regression analysis of the primary end point are shown in Table 2.

**Table 2:** Results of regression models for the study outcomes

Outcome	Fitted Mean at baseline	Coefficient before intervention ^a^	Coefficient after intervention ^b^	*p*-value	χ^2^
Primary outcomes					
I) Subjective: Self-reported vs. electronic records	1.237 ^c^	−0.305	−0.072	0.348	2.11
II) Surrogate:Pre-dialysis phosphate level (mmol/L) ^d^	1.536	− 0.088	−0.01	0.682	0.77
Secondary outcomes					
Systolic blood pressure (mmHg)	137.154	−0.623	−2.576	0.083	4.98
Low-density lipoprotein (mmol/L) ^e^	1.666	−0.754	−0.094	0.096	4.69

^a^ The coefficient of the slope of the fitted line presenting months before the intervention, ^b^ The coefficient of the slope of the fitted line presenting months after the intervention, ^c^ Mean differences in the number of oral medications between self-reporting and electronic records at baseline, ^d^ The normal range for phosphate levels is 0.8–1.44 mmol/L, which is the target level for hemodialysis patients, ^e^ Low density lipoprotein levels for patients who received lipid-lowering agents (statins)

**Fig. 5:**
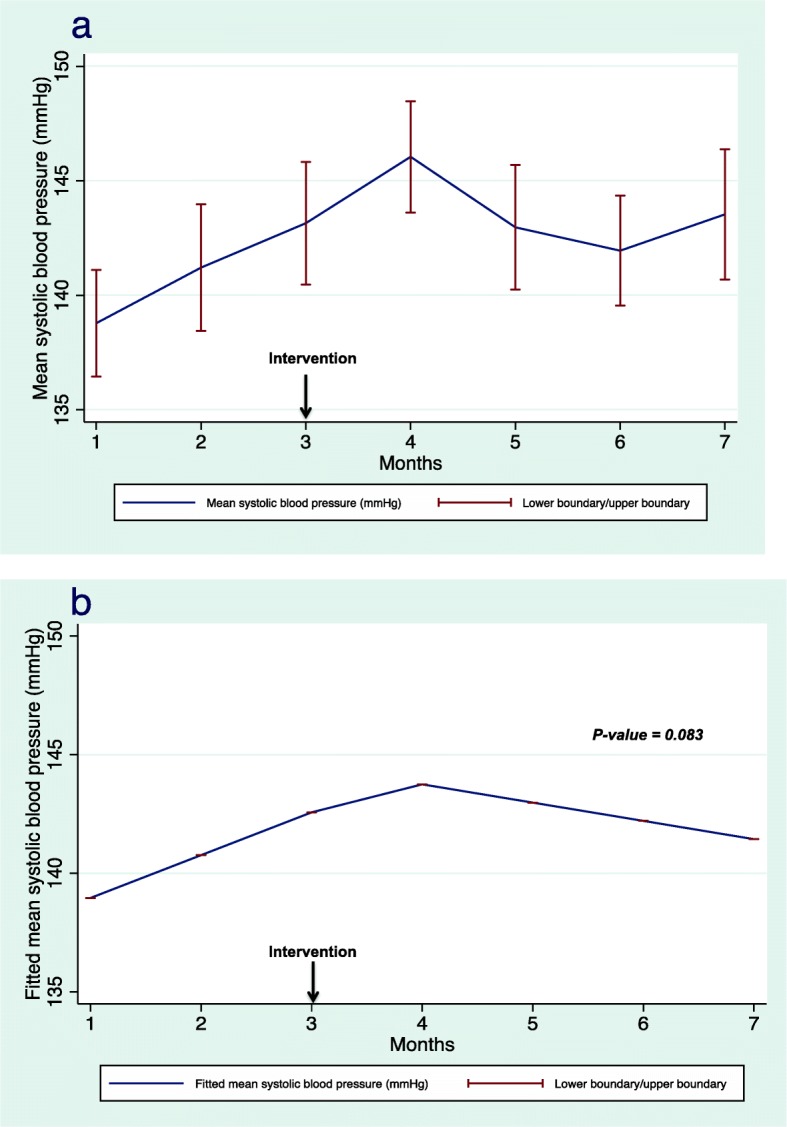
Mean (**a**) and fitted mean (**b**) systolic blood pressure. The fitted mean resulting from linear mixed regression analysis demonstrating the fitted mean before and after the intervention

**Fig. 6:**
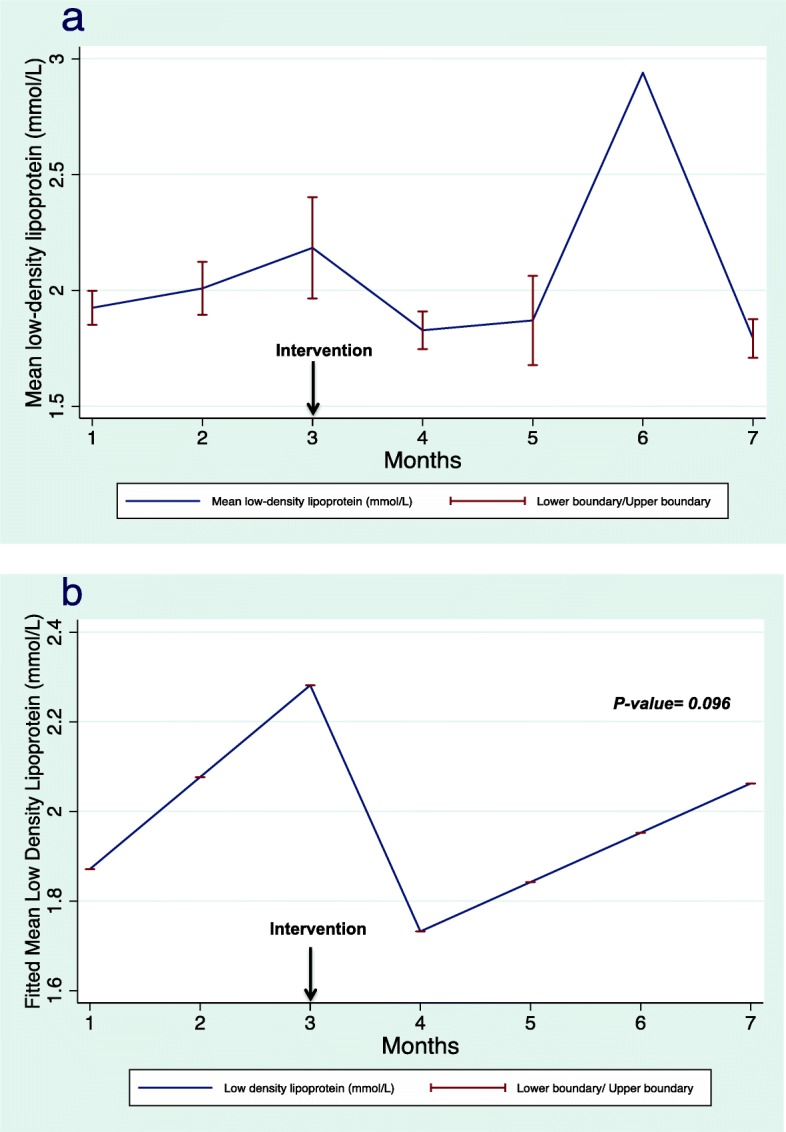
Mean (**a**) and fitted mean (**b**) low-density lipoprotein levels. The fitted mean resulting from linear mixed regression analysis demonstrating the fitted mean before and after the intervention

Regarding the secondary outcomes, mean SBP declined after the intervention, however, the results were not statistically significant (*p* = 0.083). Figure 5a illustrates the change in mean pre-HD SBP, whereas Fig. 5b demonstrates the fitted mean differences before and after the intervention. We were unable to use a linear mixed regression model to estimate the mean change in HbA1c levels because we had few observations in patients with diabetes. In addition, the mean LDL levels among patients receiving lipid-lowering agents (statins) demonstrated a statistically non-significant decrease after the intervention (*p* = 0.096). Figure 6a illustrates the change in the mean difference in LDL levels and Fig. 6b demonstrates the fitted mean differences before and after the intervention. The results of regression analysis of the secondary end points are presented in Table 2.

**Fig. 7:**
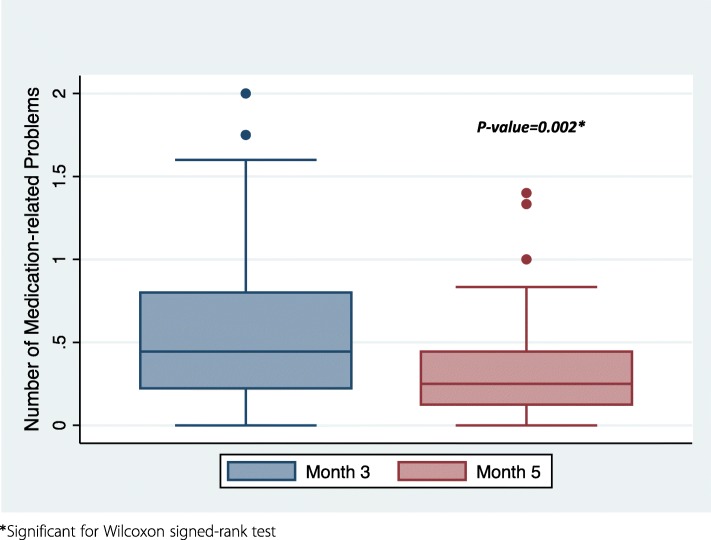
Box plot of Median number of medication-related problems

**Fig. 8:**
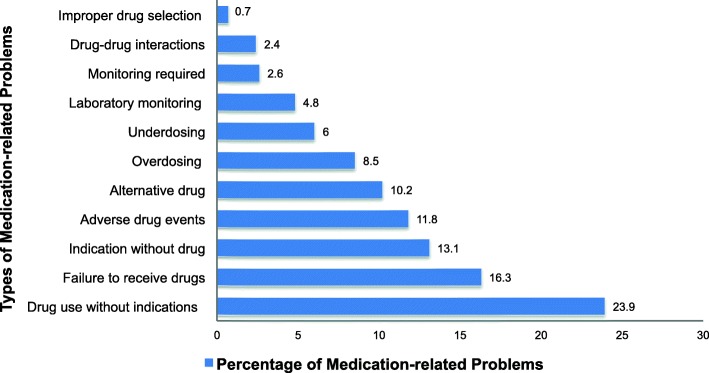
Frequencies and types of medication-related problems

The total number of MRPs identified during the study period was 421. The prevalence of MRPs at Month 3 was 44.9% (95% CI: 40.4–49.3), which decreased to 29.8% (95 CI%: 25.6–34.3) at Month 5. Additionally, the median number of MRPs per prescribed medication declined significantly from 0.44 (IQR: 0.22–0.8) at Month 3 to 0.25 (IQR: 0.13–0.44) at Month 5 (*p* = 0.002) as demonstrated by the box plot in Fig. 7. Drug use without indication was the most common MRP identified (23.9%), followed by failure to receive medications (16.3%), and indication without treatment (13.1%). Further details on the types and frequencies of MRPs are provided in Fig. 8.

The total number of interventions during the study period was 304, of which 93% were accepted. There were 43 referrals to other health-care specialties for specific therapeutic interventions. The reason for the rejection of therapeutic interventions in most of these individuals was the physician’s assessment that the benefits of continued use of some of the medications outweighed the risks.

## Discussion

In our study, patient-centered pharmacist care led to an improvement in the fitted mean difference between self-reported medication use and medications records at the EHRs as documented by electronic prescribing and a reduction in serum phosphate levels after the intervention, which are proxies for improved adherence; however, neither measure reached statistical significance. Our findings are consistent with those of a study conducted in Iran involving 86 patients requiring HD, in which clinical pharmacists adjusted medications based on laboratory results without patient interview over the course of 6 months [24]. The study aimed to meet the goals of the National Kidney Foundation Kidney Disease Outcomes Quality Initiative for the management of anemia and mineral bone disorders [24]. Although the median baseline phosphate level of 2.3 (IQR: 1.8–3.3) mmol/L was reduced to 2.1 (IQR: 1.5–3.2) mmol/L at the end of the study, the change was not statistically significant (*p* = 0.17) [24]. However, the median baseline phosphate level of our participants was much lower at 1.45 (IQR: 1.15–1.8) mmol/L. Furthermore, the median baseline phosphate level of our participants was slightly above the normal range at our institution (0.8–1.44 mmol/L), which may explain the small changes noted after the intervention. Finally, the negative findings in our setting may be partially explained by the fact that the study intervention was patient-centered care: at our HD unit, a clinical pharmacist and a dietitian already attend multidisciplinary monthly rounds and provide counseling for patients regarding their medications and dietary phosphate restriction.

However, in a systematic review that assessed the effects of educational or behavioral interventions on adherence to phosphate control among patients requiring HD [33], only four of 18 studies assessed the impact of the interventions on medication use, whereas the remainder assessed the impact of the interventions on diet alone or medications and diet combined. The eight studies included in the meta-analysis demonstrated that educational and behavioral interventions lead to a reduction in the mean phosphate level of − 0.23 mmol/L (95% CI: − 0.37 to − 0.08). These findings differ from the mean fitted change in phosphate levels post-intervention of 0.01 mmol/L (*p* = 0.68) observed in our study. Furthermore, another systematic review that assessed nonadherence to medications in patients requiring HD noted that serum phosphate level was assessed in 25% of the included studies. However, it may not be an ideal objective tool for assessing pharmacoadherence in patients requiring HD because it is influenced by many nonpharmacologic factors, such as diet and HD prescription [3]. Although all of our patients were dialyzed through high-flux membranes and some of them received few sessions of hemodiafiltration but we did not collect data regarding HD prescription. In addition, low phosphate level can be attributable to malnutrition or low phosphate intake, and does not necessarily reflect adherence to phosphate binders.

Our study did not demonstrate a significant change in pre-HD SBP. This is consistent with a study that showed that collaborative physician–pharmacist care did not result in a reduction in pre-HD SBP compared with standard care in 56 patients requiring HD after 6 months of study [34]. However, this study showed a significant decline in mean weekly home blood pressure readings (≤135/85 mmHg), which provides new insights into the control of blood pressure in patients requiring HD [34]. Our study did not demonstrate any significant changes in serum LDL before and after the intervention, which might be explained by the short duration of the study and that the levels of serum LDL were at steady state after the initial reduction of LDL from each patient’ s baseline. Alternatively, it may be due to low numbers of observations as only 29 patients who were analyzed out of 32 patients who received lipid-lowering agents (statins) had a legitimate indication to adhere to statins for secondary prevention of cardiovascular events. In addition, a recent meta-analysis pointed that LDL reduction did not lead to significant reduction in cardiac mortality or improve overall survival in dialysis patients despite reducing cardiac events, which require prolonged study period to assess [35].

Our finding of a statistically significant decline in the median number of MRPs per prescribed medication from Months 3–5 is consistent with the results of other studies. For example, a recent study reported that trained pharmacists were able to reduce the mean number of MRPs per patient from 2.16 to 1.6 after 1 year at six clinics treating patients with stages 3 and 4 chronic kidney disease [36]. In addition, a pooled analysis of seven studies that assessed MRPs in 395 patients requiring HD for an average of 3 months demonstrated a decline in the number of MRPs after each interview with the pharmacist based on regression analysis of the number of MRPs (*p* = 0.02) [10]. Improper drug selection and drug–drug interactions were the least frequently identified MRPs both in our study and in the pooled analysis [10]. However, drug use without indication (23.9%) was the most frequently detected MRP in our study leading to polypharmacy, which is a highly prevalent problem that we examined earlier in our population [37]. Therefore, there is a crucial need for the continuous comprehensive interview of medications for patients requiring hemodialysis to identify frequently prescribed drugs without indication and to implement deprescribing tools to overcome this problem [38]. In contrast, in the pooled analysis, the most frequently identified MRP was laboratory monitoring required (23.5%) [10].

Failure to receive medications was the second most common MRP identified in our population (16.3%), compared with indication without treatment (16.9%) in the pooled analysis [10]. These differences in the types and frequencies of MRPs may be attributable to differences in prescribing patterns, populations, insurance, and access to health-care settings between the studies.

Our study has several limitations. First, it was conducted over a short time period, and many of the therapeutic interventions conducted by the clinical pharmacist require a prolonged period of follow-up and sustained patient-centered pharmacist care to produce real changes such as LDL and glycosylated hemoglobin A1c with limited impact on future clinical outcomes. Second, although our intervention was based on MTM and MI, which are patient-centered approaches, we did not address other psychosocial factors or evaluated disparities in health literacy, which are reported to have a significant impact on pharmacoadherence [39]. Third, we did not conduct any formal assessment of depression in our population, which is the most important predictor of nonadherence to medications in patients requiring HD [40]. These unmeasured psychosocial and depression-related factors may have led to residual confounding. Fourth, our study presents a single-center experience with a small sample size, which limits its generalizability to similar health-care systems, populations, and prescribing patterns.

However, this study has several strengths. First, the quasi-experimental, interrupted time series design for the pre–post assessment of an intervention is useful because patients can act as their own controls to adjust for potential confounders and guard against regression to the mean. Second, the linear mixed random segmented regression model is a robust analysis for the detection of changes in the slope before and after intervention, and represents a useful tool for handling missing data with repeated measures. Third, we utilized combined subjective and objective methods for the assessment of adherence to increase the precision of the assessment of outcomes. Fourth, our pilot study presents early insights into the crucial role of patient-centered pharmacist care using MTM and MI in the reduction of MRPs due to the high acceptance rate for the therapeutic interventions and provides a structured tool for medication review in patients requiring HD. Finally, despite the statistically non-significant findings for improving adherence in the primary outcomes, we believe that the whole process of comprehensive interview using concepts of MTM and MI had a significant clinical impact on optimizing medication regimen in a patient-centered approach and therefore shall improve adherence and subsequently clinical outcomes. Future long-term, prospective, randomized, multicenter studies should address the multidimensional nature of pharmacoadherence in patients requiring HD and determine the best model incorporating MI for improving adherence. Further studies should focus on assessing the possible interactions between patient-centered pharmacist care and dietary and psychosocial factors to improve the adherence to medications of patients requiring HD.

## Conclusions

Patient-centered pharmacist care did not achieve significant changes in pharmacoadherence in patients requiring HD. However, its clinical utility as a tool to identify and mitigate MRPs in these patients is indisputable.

## Data Availability

Data is available upon request from the corresponding author.
